# Italy’s Political Turmoil and Mario Draghi’s European Challenges

**DOI:** 10.1007/s10272-021-0958-9

**Published:** 2021-04-06

**Authors:** Mario Pianta

**Affiliations:** Faculty of Political and Social Sciences, Scuola Normale Superiore, Palazzo Strozzi, Piazza Strozzi, 50123 Firenze, Italy

## Abstract

Italy’s economy is not expected to return to pre-pandemic levels before the first half of 2023, and an early return to budgetary constraints would be disastrous.

In 2021, the pace of Italy's political change has again accelerated. During the previous year, Italy was the first European country to be hit by the COVID-19 pandemic, an emergency that was faced by the second government of Giuseppe Conte, supported by the Five Star Movement (M5S), the Democratic Party (PD), a small left party, and Matteo Renzi's moderate group Italia Viva. Renzi distanced himself from the government in December 2020, leading to its fall on 13 February 2021. Mario Draghi, the widely respected former governor of the European Central Bank (ECB), was quickly called in to establish a 'national unity' government, which is now supported by all major parties with seats in key ministries – with the exception of the 'post-fascist' Fratelli d'Italia and of the small left party Sinistra Italiana. The Draghi government was expected to be more in line with the new Biden administration in the US and with the economic and financial mainstream. Greater unity and stability was anticipated in general, but in fact, Italy's fragile political system experienced the opposite.

All parties have undergone major turbulence. Matteo Salvini's Lega carried out a drastic U-turn from its extreme-right, anti-European populism and lent its support to Draghi, ending its political isolation and returning to power just in time to influence the distribution of Next Generation EU investment towards Northern Italy's business base of Lega.

Berlusconi's Forza Italia is increasingly divided between the moderate pro-Draghi wing and those emphasising the centre-right coalition with Lega and Fratelli d'Italia. Such a coalition – in spite of differences over Draghi – remains the favoured political alignment of the three parties.

The Five Star Movement was deeply divided in the vote for the Draghi government, with the leadership pressing for support, against the opposition of grassroots groups; members of parliament who did not vote for Draghi were expelled. After much confusion, Giuseppe Conte agreed to become the new leader of the Five Star Movement, bringing his large popularity to the rescue of an ailing party, which is now shedding its populism to embrace a government role and a 'centre-left' political agenda.

A similar political void at the top emerged in the Democratic Party. Its leader Nicola Zingaretti resigned over the internal squabble between the neoliberal wing – associates of Matteo Renzi who did not follow him in the split of Italia Viva – and the other party factions. The appointment as party leader of Enrico Letta – the former Prime Minister who was ousted by Matteo Renzi in 2014 – has given new energy to a party that has lost much of its working class base and has been unable to recover, gaining only 18% of the vote in the 2018 political elections under Renzi's leadership (in 2019 he split to form Italia Viva).

## Italy's turbulence

The very large parliamentary support for the Draghi government conceals a fractured political landscape; Italy's turmoil is likely to continue as a result of three main factors. First, the political crisis is here to stay. The 'bipolar' system that was forced on Italian politics in the 'Second Republic' after 1994 collapsed in the 2013 and 2018 elections with the populist success of the Five Star Movement and Lega. While the centre-right and the PD-M5S coalitions still provide the background of Italy's political stage, efforts to create a centrist neoliberal force with a clear connection to Draghi are in full swing with competing projects – including the one by Renzi – aiming to occupy such a political space. So far, Draghi is keeping parties at a distance and has barely made a public speech after the parliamentary votes. His government – with technocrats in key economic ministries – provides a temporary reprieve, but no stabilisation of the political system. The paradox is that Draghi's arrival – expected to bring competence and political strength – has in fact increased the instability of political alignments. Moreover, Draghi's eyes are on Italy's presidential election scheduled for January 2022, where he is expected to draw large cross-party support. A major realignment could emerge prior to the 2023 elections.

Second, the demise of populism is evident. M5S and Lega have shifted to 'responsible' political choices, although

Open Access funding provided by ZBW – Leibniz Information Centre for Economics.

**Mario Pianta**, Scuola Normale Superiore, Florence, Italy.

Salvini's rhetoric continues. Their coalition in Conte's first government in 2018 failed to reshape the political system, barely lasted a year, achieved little and deeply divided the country. As the largest forces in parliament, M5S and Lega progressively abandoned their anti-politics platform and turned into institutional players. The extreme-right, anti-European banner is now raised by Fratelli d'Italia. The centrist shift of the M5S has widened the political void on left and green issues, where no significant political force is emerging.

Third, the economic crisis is dramatic. In 2020 Italy's GDP fell by 8.9% (Istat, 2021); in the twelve months of the coronavirus pandemic 600,000 jobs were lost – mainly among fixed-term workers and the self-employed. The ban on layoffs of permanent workers has been extended to June 2021, when job losses could skyrocket. The previous government had confronted the crisis with €110 billion of deficit spending, of which €60 billion went to across-the-board subsidies to firms and the rest was mainly for household income support.[1] Draghi has announced plans to reorganise such measures, but will likely be forced to continue with similar emergency policies. Likewise, in the public health measures for confronting the pandemic, a continuity can be found with the measures of the Conte government, with the vaccination plan now in the hands of an army General.

These three factors are deeply rooted in Italian problems, but have a close connection to the European dimension, where key political decisions are made.

## Draghi's Europe?

At home, the success of Mario Draghi's government will be measured by his ability to control the pandemic, address the economic crisis and obtain the €209 billion of Next Generation EU funds allocated to Italy. But much of the solution to these challenges lies in Brussels. In fact, Draghi's success will mainly depend on his ability to change Brussels' political agenda and revise the economic rules of the Union, providing Italy with the policy space that is needed to confront its complex crises. Draghi is facing three challenges in Europe that are likely to be much more difficult than forming his government in Rome.

### European fiscal policy

With the COVID-19 emergency, Europe activated the "general escape clause" of the Stability and Growth Pact, suspending the obligation of governments to move towards balanced budgets. In the early months of 2020, Italy played a major role – with Prime Minister Giuseppe Conte, Economy Minister Roberto Gualtieri and EU Commissioner Paolo Gentiloni – in this decision, as well as in the launching of Next Generation EU as the first instrument of a common fiscal policy in Europe.

On 3 March 2021, the European Commission decided to extend the suspension of the Stability and Growth Pact until the end of 2022, allowing the government deficit spending needed to confront a continuing crisis. Italy's economy is not expected to return to pre-pandemic levels before the first half of 2023, and an early return to budgetary constraints would be disastrous.

The thornier question is the rewriting of the EU's fiscal rules. Although this debate is slowly getting under way,[2] it will not take off before the 2021 elections in Germany. With Angela Merkel leaving office this autumn, Draghi has a real opportunity to step in, frame the debate in novel terms and redress some of the worst mistakes of Europe's economic Union, starting with the Maastricht rules on public budgets and debt. In his inaugural speech, Draghi discussed the "irreversibility" of the euro and emphasised "the prospect of an increasingly integrated European Union that will lead to a common public budget capable of sustaining countries in periods of recession" (Presidenza del Consiglio dei Ministri, 2021). Draghi's plans for the new fiscal capacity of the EU should be presented as soon as possible and should offer a clean break with the austerity policies of the past.

At the very least, there is a need to make Next Generation EU a permanent tool of EU policies, financed with Eurobonds and supporting appropriate public spending where the Union needs it most. On the revenue side, there is a major need for fiscal harmonisation in order to avoid tax competition within the EU and for new EU-wide taxes on digital platforms, multinational corporations, financial transactions, carbon emissions and imports, etc.

### Public debt

In his *Financial Times* article, Draghi (2020) wrote that, in the face of the pandemic, "it is already clear that the answer must involve a significant increase in public debt". In 2020, its increase has been generalised in Europe and a significant part – about a quarter for some countries – of the public debt is now held by the European Central Bank. While current ECB monetary policies – near-zero interest rates and quantitative easing – have made debt

1 See Pianta et al. (2020) for an assessment of Italy's economic policy in the COVID-19 crisis.

2 See the proposals of German Finance Minister Olaf Scholz on the 'debt brake' (Hall, 2021); and Blanchard et al. (2021).

financing manageable for governments, a structural solution has to be devised, the sooner the better. A key proposal is to "freeze" the debt in the hands of the ECB, transforming it into perpetual zero-interest bonds. In an interview on 14 November 2020 with the Italian daily *La Repubblica*, the President of the European Parliament David Sassoli declared that debt cancellation is "an interesting working hypothesis, to be reconciled with the fundamental principle of debt sustainability" (D'Argenio, 2020). An appeal from over 100 European economists, including Thomas Piketty, has recently called for the cancellation of public debt in the hands of the ECB, asking governments to allocate the same amount of resources to new social and environmental investments (Le Monde, 2021).

### Industrial policy

Due to the pandemic, Brussels suspended the ban on state aid to businesses; all governments – Germany more than any other country – offered subsidies, tax breaks and public capital to the companies most affected by the crisis. This state aid suspension is temporary: If it were reintroduced, millions of European companies would go bankrupt. Even the so-called frugal countries are worried: the Danish Minister of Industry – together with Austrian and Czech ministers – has asked Brussels to raise the limit of subsidies to companies (€800,000) and of the compensation allowed so far (currently €3 million; Joergensen, 2021).

The European meetings of economic ministers on 15-16 February 2021 confirmed the need to support firms that will be viable in the post-pandemic economy. This is in line with the report of the Group of 30 (2020), an international body of financiers and academics chaired by the former governor of the Indian Central Bank Raghuram Rajan and by Draghi himself, which has provided indications to governments on how to move from general support to business towards more targeted measures, ensuring the prudent use of limited public resources and allowing market forces to manage "the pace of the needed creative destruction".

What will be Draghi's agenda on policies for a post-pandemic production system? Here again, a radical rewriting of European rules is needed. Public intervention orienting and supporting business choices should no longer be seen as a "distortion" of the market, allowed as an exception only. Europe needs to institutionalise an industrial policy charting a new growth trajectory based on environmentally sustainable economic activities with a high content of knowledge, technology and quality of work, a policy that should strive, at the same time, to reduce social and territorial disparities among European regions.

Industrial policy has indeed made a comeback in Europe's agenda. Germany and France are pushing their plans in key fields – from high technology to electric cars – with the aim of strengthening industrial sovereignty and autonomy in strategic areas (Pianta et al., 2020; Federal Ministry for Economic Affairs and Energy and Ministry for the Economy and Finance, 2019, 2021). Vaccines are a key case, and former president of the EU Commission Romano Prodi (2021) has argued that governments should organise and finance the production of COVID-19 vaccines in "the largest possible number of firms" in all countries.

The opportunity to move in this direction is there and includes investments funded by Next Generation EU that could be a first step in a new trajectory of digital-age, environmentally sustainable growth. The key question is whether Draghi will pick up this challenge for rebuilding the economy of a post-pandemic Europe.

### Long-term reconstruction

In his inaugural speech, Draghi stated that

> the government will have to protect workers, all workers, but it would be a mistake to protect indifferently all economic activities. Some will have to change, even radically. And the choice of which activities should be protected, and which ones should be gradual change is the difficult task that economic policy will have to face in the coming months. (Presidenza del Consiglio dei Ministri, 2021)

However, he gave no indication of the objectives of his government's choices, nor of the policy tools that could stimulate research, investments, productions and employment. He did not outline the role that the country should have in key industries. In his speech, Draghi never mentioned the term "industrial policy". This is worrying in the context of Italy's deepening crisis. These problems did not start with the coronavirus pandemic; the country is facing the legacy of a decade-long recession. The crisis of 2011-2014 wiped out 200,000 firms and 800,000 jobs; compared to 2007, in 2019 the economy still had 5% fewer hours worked and the industrial production index had lost almost 20% (Cresti et al., 2020). The need for reconstruction would have existed even without the pandemic. Moreover, the pandemic has highlighted other weaknesses in the Italian economy: the extent of precarious work, the incomplete protection of incomes and the distortions of the welfare system.

For Italy, the opportunity provided by the resources of Next Generation EU is indeed crucial to rebuilding production capacities and starting a new growth trajectory. But a broader industrial policy framework would be required, with clear objectives and novel policy tools. The latter could include a public investment agency, a holding company concentrating public shareholdings and a public investment bank capable of taking over and assisting declining companies and launching new ventures in priority fields.[3]

There are major obstacles – economic, political and institutional – to overcome on Italy's road to recovery. The first one is the unending political instability described above. A successful industrial policy for rebuilding the economy should be a clear government priority based on a long-term vision with a strong political commitment.

Secondly, the industrial policy strategy should be shared by companies, the trade unions, workers, civil society and public opinion, turning it into a theme above the fray of short-term conflicts. A broad consensus should emerge on a new balance between market activities and public intervention, between capital and labour, and among environmental, social and economic priorities, with a wide distribution of expected benefits, focusing on employment creation, reduction of job insecurity, fewer territorial, social, gender and health inequalities[4].

Finally, the implementation of industrial policy requires a renewed public administration, with greater capabilities, higher efficiency, ensuring transparency and democratic participation.

It is not easy for these three conditions to be fulfilled. However, after the COVID-19 pandemic, the alternative to a reconstruction of this type would simply be a worsening of the decline that has marked Italy in recent decades. And a failure of Italy in this effort would also be a failure of Europe.

The challenges that Draghi is facing in Brussels are parallel to the ones he is confronting in Rome. They amount to an opportunity for change that has appeared all but impossible for decades – that is, the political space for rewriting the rules of the European Union. As head of the ECB, Draghi was bound to respect the Treaties and government policies; yet he managed to overturn the conservative monetary policy he inherited in 2011 from Jean-Claude Trichet. Now, as leader of the Italian government, Draghi has the opportunity to give Europe the coherence between fiscal and monetary policy that it has been lacking so far. But the political opportunity before us is grander than that – the troubled neoliberal past of austerity could be replaced by a healthier, smarter, more sustainable and more equal Europe.

3 See Cresti et al. (2020) for our proposal for an industrial policy.

4 Such an agenda was proposed by the Sbilanciamoci! (2020) campaign.
